# Going beyond histology. Synchrotron micro-computed tomography as a methodology for biological tissue characterization: from tissue morphology to individual cells

**DOI:** 10.1098/rsif.2008.0539

**Published:** 2009-03-25

**Authors:** Rolf Zehbe, Astrid Haibel, Heinrich Riesemeier, Ulrich Gross, C. James Kirkpatrick, Helmut Schubert, Christoph Brochhausen

**Affiliations:** 1 Institute of Materials Science and Technologies, Technische Universität Berlin, Englische Strasse 20, 10587 Berlin, Germany; 2 GKSS Research Centre at DESY, Petra III, Notkestrasse 85, 22607 Hamburg, Germany; 3 BAM, Federal Institute for Materials Research and Testing, Division I.3, Richard-Willstätter-Strasse 11, 12489 Berlin, Germany; 4 Biomaterials Laboratory, Charité University Medicine Berlin, Aßmannshauser Strasse 4-6, 14197 Berlin, Germany; 5 REPAIRlab, Institute of Pathology, Johannes Gutenberg University, Langenbeckstrasse 1, 55101 Mainz, Germany

**Keywords:** cartilage, chondrocyte, synchrotron micro-computed tomography, histology, scanning electron microscopy, three-dimensional imaging

## Abstract

Current light microscopic methods such as serial sectioning, confocal microscopy or multiphoton microscopy are severely limited in their ability to analyse rather opaque biological structures in three dimensions, while electron optical methods offer either a good three-dimensional topographic visualization (scanning electron microscopy) or high-resolution imaging of very thin samples (transmission electron microscopy). However, sample preparation commonly results in a significant alteration and the destruction of the three-dimensional integrity of the specimen. Depending on the selected photon energy, the interaction between X-rays and biological matter provides semi-transparency of the specimen, allowing penetration of even large specimens. Based on the projection-slice theorem, angular projections can be used for tomographic imaging. This method is well developed in medical and materials science for structure sizes down to several micrometres and is considered as being non-destructive. Achieving a spatial and structural resolution that is sufficient for the imaging of cells inside biological tissues is difficult due to several experimental conditions. A major problem that cannot be resolved with conventional X-ray sources are the low differences in density and absorption contrast of cells and the surrounding tissue. Therefore, X-ray monochromatization coupled with a sufficiently high photon flux and coherent beam properties are key requirements and currently only possible with synchrotron-produced X-rays. In this study, we report on the three-dimensional morphological characterization of articular cartilage using synchrotron-generated X-rays demonstrating the spatial distribution of single cells inside the tissue and their quantification, while comparing our findings to conventional histological techniques.

## 1. Introduction

Our morphological understanding of the human body with respect to pathological anatomy was probably most influenced by Morgagni (1761), Bichat (1802) and Virchow (1859) cumulating in the development of modern histology as one of the principal methodologies for the understanding of the structural organization of tissue in health, disease and regeneration. Although Bichat achieved considerable knowledge without using microscope equipment, since that time, microscopes in combination with tissue-specific staining protocols have been considered to be the principal tool in histopathological research and diagnostics.

Virchow (1859) established a concept that is focused on the cellular level of living processes and stated that all physiological disorders are based on morphological changes in organs, tissues and cells. Since that time, further efforts have been made in the optical components of light microscopes and in histological techniques.

The major disadvantage in light microscopy is the limited penetration depth of optical wavelengths in biological tissues. Consequently, the assessment of three-dimensional tissue structures is difficult to achieve. For small spatial structures, confocal microscopy (structure depth <100 μm) and multiphoton microscopy (structure depth <400 μm) were developed and allow excellent imaging results (Minsky 1961; Cahalan *et al.* 2002). Samples that are larger can only be investigated using serial sectioning, a technique which is both time consuming and which requires precise alignment of probably deformed subsequent serial sections (Braverman & Braverman 1986; Bussolati *et al.* 2005), while ultimately destroying the specimen.

Furthermore, the majority of histological procedures require fixation and dehydration resulting in significant physico-chemical alterations of the tissue with relevant consequences for structural organization and composition of the material. In this context, Burck (1982) has estimated the volumetric shrinkage due to formalin fixation to be approximately 3–6 vol%, while the dehydration with series of graded ethanol may contribute with up to 20 vol% to the sample shrinkage (Romeis 1989). In the case of calcified tissue such as bone, it is often necessary to remove calcium by acid dissolution or complexation with ethylenediaminetetraacetic acid (EDTA). Taking all these alterations together, Nissl (1894) concluded already in the nineteenth century that histological imaging does not display the real native tissue morphology but an image equivalent to the natural state.

In contrast to optical microscopy, electron microscopy has extended the morphological understanding towards subcellular details with a resolution on the nanometre scale. However, it is not able to resolve volumetric details in larger tissue volumes. Scanning electron microscopy (SEM) resolves well the topography but offers no volumetric details inside a sample due to the limited penetration depth of electrons. Although, SEM in conjunction with a focused ion beam (FIB) for slicing can be used similar to serial sectioning allowing for the three-dimensional reconstruction of a sample, according to Schmidt *et al.* (2007) or Martinez *et al.* (2008). Alternatively, transmission electron microscopy (TEM) imaging can be used for the reconstruction of small, highly detailed sample volumes by tilting ultrathin slices (De Rosier & Klug 1968). For both methods (FIB/SEM and TEM), it becomes increasingly important to introduce specific stainings to better differentiate between structures in the sample. Typically, these stainings are based on heavy metals such as osmium tetroxide (Marton 1934) and have opened a new dimension in our structural view of physiological and pathophysiological processes.

All these preparational disadvantages may apply to X-ray-based imaging methods as well. X-rays further show negligible refraction that results in poor absorption contrast in biological tissues; therefore, metal stains are commonly used to better differentiate between structures. The invaluable benefit of X-ray imaging is the high sample penetration that is coupled with a theoretical resolution between that of optical wavelengths and electrons. Of course, the latter does not apply for set-ups relying on the conversion of X-rays into visible light through scintillators, which is often the case in tomographic experimental set-ups.

X-ray-based tomography was pioneered by Cormack (1973) and Hounsfield (1973) using X-ray tubes. With the advance of synchrotrons, another source for X-rays was established outperforming those generated in X-ray tubes. Synchrotron-produced X-rays offer a high photon flux over a large range of X-ray energies, high brilliance, small angular beam divergence, high level of polarization and coherence, low emittance and the possibility for monochromatization (Dilmanian 1992; Bonse & Busch 1996). Owing to these advantages, synchrotrons can principally achieve qualitatively better and faster measurements than X-ray tubes with an overall similar experimental set-up (Bernhardt *et al.* 2004).

Synchrotron radiation-based micro-computed tomography (SR-μCT) requires an experimental set-up as shown in figure 2, and consists of a high-precision sample stage (rotation table combined with translation tables perpendicular to the beam), with a sample holder, a scintillator and subsequent light optics with a charge-coupled device (CCD) camera. The mechanical stability of the sample movement (translation and rotation) and also the quality of the scintillator material and the number of recorded angular projections are key experimental conditions to achieve tomographic images, with a true micrometre resolution. The methodology to obtain the three-dimensional reconstruction of the projection images uses a mathematical approach that is commonly referred to as the Radon transform (Radon 1917; Kak & Slaney 1988).

The use of SR-μCT in life sciences dates back to the works of Feldkamp *et al.* (1989) who pioneered this technique to analyse trabecular specimens.

This method has since then been significantly improved in both computer processing capabilities and experimental set-ups. Until now, we and other groups have shown that a three-dimensional quantification of cells in synthetic, low-density materials such as scaffolds is achievable by labelling with specific or unspecific metal stains enhancing the absorption contrast (Thurner *et al.* 2001, 2005; Ginty *et al.* 2006; Müller *et al.* 2006*a*; Zehbe *et al.* 2007). By contrast, homogeneous metal staining in dense samples, such as native tissues, can be difficult to achieve due to diffusion limitations of the metal stain, which might (depending on the exact chemical composition of the stain) result in highly contrasted outer regions and lesser contrasted inner regions of the sample and possibly introducing artefacts due to the resulting difference in absorption contrast of stronger and lesser stained structures. It has been reasoned that phase contrast imaging might be more suited for structures with a low difference in absorption contrast (Müller *et al.* 2006*b*). Phase contrast imaging has been further demonstrated by Cloetens *et al.* (2006) showing cellular structures in *Arabidopsis* seeds.

**Figure 1:**
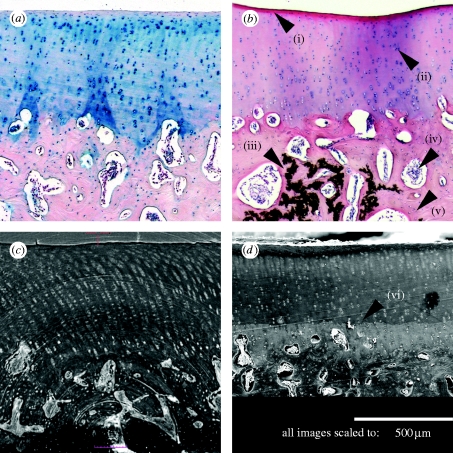
Comparison of stained histological slices with SEM and SR-μCT of the same sample at the same magnification (scale bar, 500 μm). (*a*) Histology, Hale-PAS stained; (*b*) histology, haematoxylin and eosin; (*c*) SR-μCT, minimum intensity projection of 10 subsequent stack images; and (*d*) SEM. For the description of (i)–(vi) please refer to the text.

In this work, we compare SR-μCT with conventional histology and show how histological details can be revealed in non-stained samples. Articular cartilage and the underlying subchondral bone were chosen for characterization, as this tissue features both soft and hard tissue structures with a wide range in X-ray absorption contrast (figure 1).

Articular cartilage shows a characteristic zonal structure of the extracellular matrix (ECM) components (primarily collagen II and proteoglycans), in which the cartilage cells, the chondrocytes, are embedded. It is generally divided into the following regions: surface zone; middle zone; deep zone; and subchondral bone (Mow & Lai 1976; Lambert *et al.* 1986; Buckwalter & Mankin 1997). Depending on the zonal region, the chondrocytes appear in different morphological shapes, stretched along the surface in the superficial zone, and mostly elliptical or rounded in the middle zone, where some cells form isogeneous cell groups. Chondrocytes are generally surrounded by a low-density fluidic region called lacuna. In deeper regions, the collagen fibres become more and more perpendicularly oriented to the surface resulting in a rise in stiffness. This zone becomes increasingly calcified as indicated by a structure known as the tide mark. Calcification increases in the distal direction forming the subchondral bone. Finally, the subchondral bone features a broader spectrum of cells, including osteoblasts, osteoclasts, osteocytes, fatty cells and cells attributing to the nervous and vascular system.

## 2. Material and methods

A cylindrical cartilage-bone plug was harvested from the joint of a 24-month-old cow and was immediately fixed, according to Lillie (1954), in phosphate-buffered (pH 7.0) formaldehyde (3.5%) at 4°C for 24 hours. Afterwards, it was first rinsed in water for 2 hours and then decalcified at room temperature using EDTA, according to Romeis (1989) for 4 days and daily change of EDTA. After EDTA treatment, the sample was rinsed again in water for 2 hours. Dehydration was done using a series of graded ethanol (rinsing in 70, 80, 96 and twice in 100% for 4 hours each). Finally, the sample was rinsed twice in methyl benzoate for 20 min and sectioned into two halves, before embedding in paraffin. One half was used for histological characterization using light microscopy, while the other half was used for the SR-μCT analysis and SEM/FIB.

### 2.1. Histology and SEM

The bovine tissue sample was sequentially sectioned into 20 slices of approximately 4 μm thickness followed by deparaffination and staining (haematoxylin and eosin, Leica ST 4040 autostainer). Additionally, two approximately 10 μm thick slices were prepared for manual staining with haematoxylin and eosin and Hale-PAS.

Out of 20 slices, 17 slices were actually considered for the later computer-assisted three-dimensional tissue reconstruction, as strong sectioning artefacts were found on three slices. All slices were imaged with a Leica DMRM microscope at approximately the same image region with a 5× objective (Leica N Plan) using a Leica DFC 320 digital camera for image acquisition at a combined magnification of 50×. For each imaged slice, the same settings for brightness and contrast were used. Subsequently, the images were automatically aligned using ImageJ (Abramoff *et al.* 2004) and the StackReg plug-in (Thévenaz *et al.* 1998) and were afterwards cropped, delivering a square-shaped region of interest featuring all structural details (calcified tissue, decalcified tissue by EDTA treatment and cartilage). The stack was further resampled in the direction of the *z*-axis with a resampling factor of four, to approximate the sectioning thickness (4 μm) to the *xy*-pixel size at 50× magnification (spatial resolution of 1.1 μm). After each slice, a spacing was included to indicate possible sectioning losses between two subsequent slices and missing volume information on account of the reduction to a single focal plane. The resulting image stack was rendered using VGStudio MAX (Volume Graphics, Heidelberg, Germany) to yield a three-dimensional representation of the serial sections.

SEM imaging in combination with FIB milling was performed after SR-μCT analysis using a Zeiss Cross Beam EsB 1540. The sample was previously gold sputter-coated in an argon atmosphere at 1.0×10^−2^ mbar using a Balzers SCD050. Previous to SEM imaging, the region of interest was FIB milled using a gallium source ion beam to give a completely planar (on the nanometre scale) surface.

### 2.2. Synchrotron X-ray imaging

The non-sectioned half of the specimen was deparaffinized in xylene until the sample was completely removed from the surrounding paraffin. This step was considered beneficial to increase contrast in both absorption and phase contrast imaging modes, exchanging the paraffin intermedium with air. Afterwards, the sample was stored in dry atmosphere using silica gel as a desiccant. Subsequently, SR-μCT measurements were performed at the BAMline (BAM, Federal Institute for Materials Research and Testing) at BESSY Berlin (Berlin electron storage ring company for synchrotron radiation). A detailed description of the BAMline set-up is available online (Riesemeier *et al.* 2007).

The monochromatic synchrotron radiation used in combination with a thin single CdWO_4_ crystal as a scintillator, microscope optics and CCD camera (2048×2048 pixels) arranged behind, allowed for a spatial resolution of approximately 1.6 μm. The spatial resolution was determined geometrically in projection by displacing a known test object. Owing to the non-equivalence of projection data and reconstructed data, this methodology allows only for an approximation and is probably not as exact as a determination using an edge phantom and calculation of the modulation transfer function, as described, for example, by Müller *et al.* (2002).

#### 2.2.1. Phase contrast versus absorption contrast

**Figure 2:**
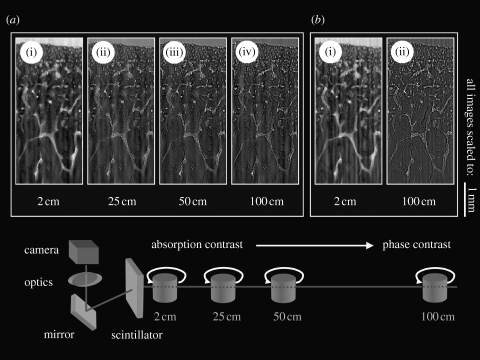
Influence of the beam energy and the distance between the scintillator and the sample resulting in the attenuation of phase contrast or absorption contrast and the impact on the visual detail. Non-decalcified histological cartilage sample embedded in epoxy resin mounted on a polymer microscope slide. Sample to scintillator distances greater than 2 cm increase the visual detail. The higher beam energy of (*b*) 15 keV compared with (*a*) 10 keV slightly increases the visual detail.

As pure absorption contrast was considered to be insufficient for the retrieval of structural details in the soft tissue part of the sample, a validation experiment for phase contrast versus absorption contrast was performed on a different cartilage specimen but from the same animal and joint, which was embedded in resin (poly(methyl methacrylate)), mounted on a polymer microscope slide and polished down to approximately 20 μm. This specimen was exposed to the synchrotron generated X-rays at different stage positions and two different X-ray energies according to figure 2.

Owing to the low thickness of the specimen, experiments were only performed in projection mode, recording a single magnified image of the sample.

#### 2.2.2. Qualitative and quantitative SR-μCT

The sample was positioned 15 cm away from the scintillator screen to permit both phase contrast and absorption contrast as detailed above. The region of interest recorded by the CCD camera was set to 2048×1500 pixels allowing for a maximum sample width of 3.3 mm. The sample was rotated in steps of 180°/1200 and was exposed to the beam at 14 keV for an exposure time of 2.0 s. Both dark-field (removal of camera-specific artefacts) and flat-field corrections (removal of beam-related artefacts) were applied to limit formation of ring artefacts during reconstruction. Persistent ring artefacts were compensated later via sinogram correction according to Boin & Haibel (2006). Reconstruction of the obtained projected images was performed using filtered back projection as described by Basu & Bresler (2000). The data were acquired in a camera-specific 16-bit RAW format, which was tone mapped to 8 bits while preserving a high contrast between all relevant tissue structures. These data were saved as a multi-image TIFF stack. The reduced data amount enabled processing on an Apple iMac Core 2 Duo (2.3 GHz, 3.3 Gbyte RAM).

**Figure 3:**
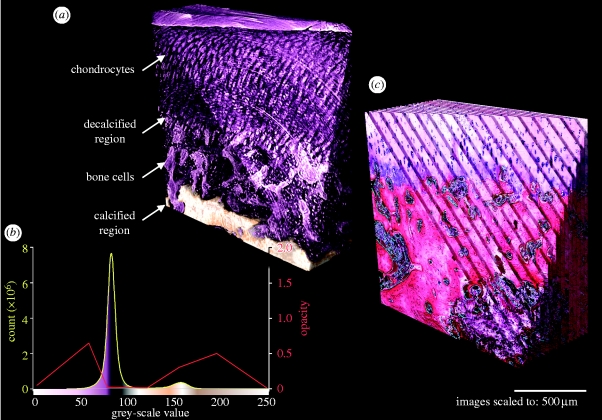
(*a*) Rendered SR-μCT data corresponding to (*b*) the applied histogram CLUT/opacity level indicating relevant structures and (*c*) rendered histological data from serial sectioning (see the electronic supplementary material, movie).

Two-dimensional sliced and three-dimensional rendered data were obtained using the software OsiriX v. 3.0 (http://www.osirix-viewer.com) and VGStudio MAX v. 1.2.1 (Volume Graphics). To further enhance the cellular distribution and to more easily distinguish between soft and calcified tissues, the histogram grey-scale values were remapped corresponding to figure 3. The depicted 16-bit colour lookup table (CLUT: pink, soft tissue; light yellow, calcified hard tissue; and white, chondrocytes/lacunae) and the applied opacity values were chosen to be visually similar to the haematoxylin and eosin-stained serial sections. An intensity minimum (black colour) was set at a grey value of 90 separating the large peak at grey value 85, while also applying an opacity minimum, resulting in high translucency of the soft tissue parts but visually preserving the cell lacunae.

The representation of a single chondrocyte inside its lacunae was achieved by digitally magnifying (10 times) and median filtering (8 pixel radius) of the cropped TIFF stack in each axial direction using ImageJ. The filtered data were rendered using OsiriX applying a CLUT, which was adapted to the slightly different grey-scale histogram. The volume of a single lacuna with its chondrocyte was demonstrated using the ImageJ ‘three-dimensional object counter’ plug-in (Müller *et al.* 2002) on the thresholded (binarized) data.

**Figure 4:**
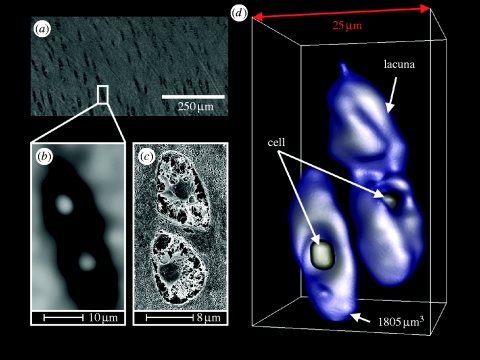
Demonstration of cellular details in the soft tissue region of articular cartilage. (*a*) Original sliced data, (*b*) 10× digitally magnified (median-filtered) region of a lacuna doublet with their chondrocytes in the centres, (*c*) correspondence with SEM/FIB and (*d*) rendering of the 10× magnified data and volume estimation for the frontal lacuna (three-dimensional reconstruction).

Quantification of the cell density was performed on the basis of the volume estimation of the single cell in its lacunae, as described above and following the steps outlined in table 1. Briefly, a cuboid region of interest was chosen, showing only the soft tissue cartilage near the surface. The grey-scale data were median filtered to remove most of the persistent artefacts. Binarization by thresholding delivered the lacunae. The threshold value of 78 was chosen to be at half of the left-hand side of the histogram peak (grey value 85), as shown in figure 3. The data were again median filtered to remove thresholding artefacts. Using the three-dimensional object counter plug-in (Bolte & Cordelières 2006) on the data delivered the total amount of lacunae and therefore the amount of cells inside the volume. The resulting histogram represents the acquired particle volumes (for objects larger than 750 μm^3^, which is half the peak volume of 1500 μm^3^, and considerably less than the calculated volume for a single cell in its lacunae of 1805 μm^3^, as depicted in figure 4 *d*). Furthermore, circularity and orientation of the cell lacunae was analysed. Briefly, fit ellipses and circularity were calculated for each lacuna in the image stack using ImageJ and its ‘analyse particles’ function. For each fit ellipse, the orientation angle and the circularity (values of 1.0 represent a circle, while a value of 0.0 indicates an elongated polygon) were recorded and plotted against each other as the density plot. The exact determination of circularity in ImageJ is according to the formula: circularity=4*π*(area/perimeter^2^).

**Table 1:** Processing steps in ImageJ for cell density quantification.

step	visual example (sliced data)	method and parameters (ImageJ)	description
1	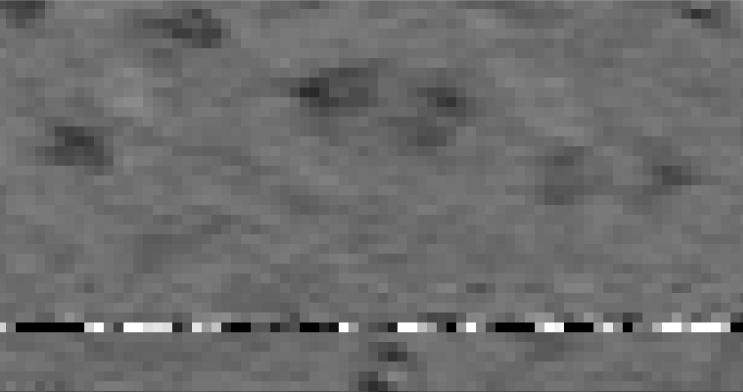	*region of interest*	
		500×500×250 (voxel) 800×800×400 (μm^3^)	a cuboid region featuring only the cartilaginous part of the entire dataset was chosen
	
2	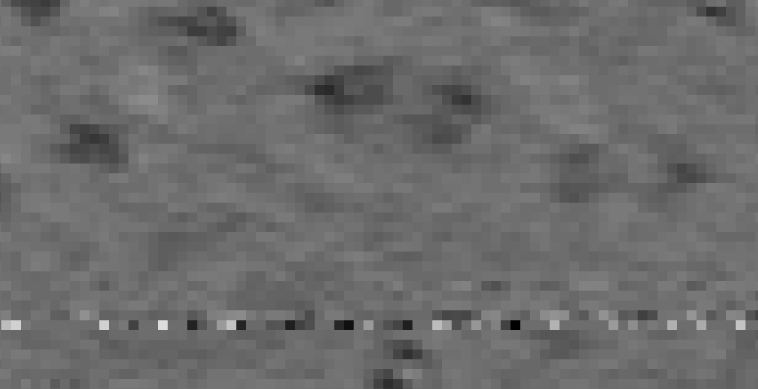	*remove outliers*	
		radius: 16 pixel threshold: 50	median filter replacing a pixel by the median of pixels in the vicinity if it deviates from the median by more than the threshold value
	
3	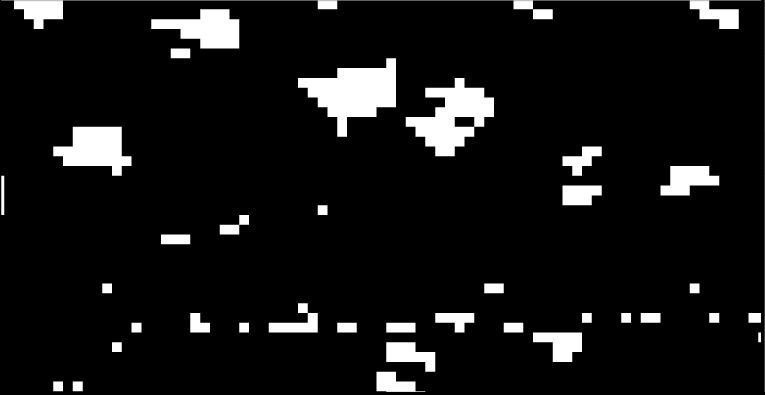	*threshold*	
		threshold: 78	image binarization by setting grey values to black and white corresponding to the threshold value
4	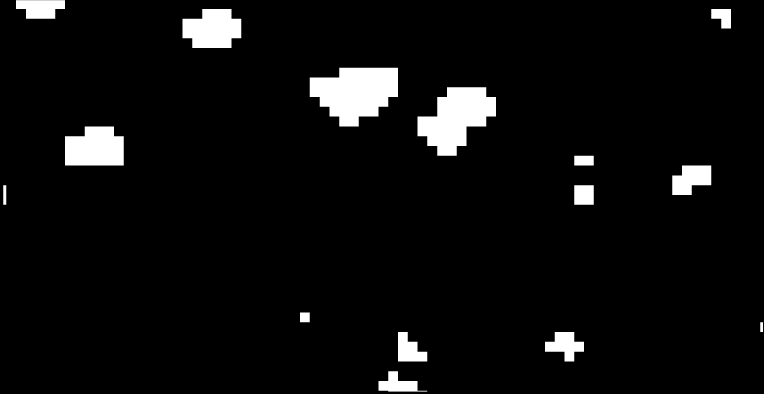	*despeckle*	
		program default	median filter replacing each pixel with the median value in its 3×3 vicinity
5	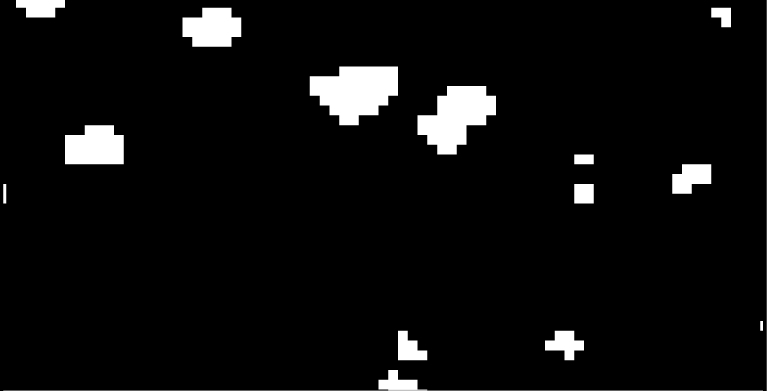	*three-dimensional object counter*	
		threshold: 128 slice: 100	ImageJ plug-in; Bolte & Cordelières (2006)
	
		voxel size: 750−62.5×10^6^

## 3. Results

### 3.1. Histology

A comparison of the different two-dimensional imaging techniques of this study is presented in figure 1. All images were scaled to the same spatial resolution, with figure 1 *a* (haematoxylin and eosin) and figure 1 *b* (Hale-PAS) demonstrating the histological tissue representation. Apart from different colorations, the major difference between these images is (iii) the missing calcification residue in the Hale-PAS stain. Figure 1 *c* displays the virtually sliced image data from SR-μCT, while figure 1 *d* shows the previously FIB-milled and SEM-imaged sample. Briefly, (i) the superficial zone features a discoloration in the histological stains due to an enrichment of proteoglycans. Beneath this zone, (ii) the chondrocytes in their lacunae stacked in columns are seen extending below (vi) the tide mark, where the soft tissue becomes calcified forming the subchondral bone. Even after EDTA treatment, necessary for histological sectioning, some calcified regions are persistent, resulting in (iii) the presence of a coarse dark network of crystalline hydroxyapatite. Throughout the subchondral bone, different cell types can be observed and attributed to (iv) clustered fatty cells and (v) single osteoblasts/osteocytes.

### 3.2. Phase contrast versus absorption contrast

To identify a sample stage position relative to the scintillator screen allowing for both phase contrast and absorption contrast, we found stage positions farther than 2 cm away from the scintillator screen to be greatly beneficial for the imaging of structural details.

These results appear to be consistent with the work of Cloetens *et al.* (2006), where a short distance to the scintillator favours absorption contrast while far away distances favour phase contrast.

Furthermore, for the given specimen, a combination of higher beam energy (15 keV) and a long distance (100 cm) offered best results concerning structural details. So as not to over-attenuate phase boundaries, an intermediate distance of 15 cm and a beam energy of 14 keV were chosen for tomographic experiment described below.

### 3.3. Qualitative SR-μCT

The tomographic data with an effective voxel size of 1.6 μm show both contrast modes due to the highly coherent and monochromatic beam. Furthermore, the beam energy of 14 keV allowed penetration of both soft tissue and hard tissue zones, while achieving good absorption contrast in both tissue regions. Additionally, dedicated sample positioning 15 cm away from the scintillator screen favoured the phase contrast mode.

Consequently, structures with large differences in density or atomic mass, in this case calcified tissue and non-calcified tissues, are well imaged on account of differences in absorption contrast. Nevertheless, structures with a small difference in their density/atomic mass, such as ECM and cells, also image well due to the attenuation of phase boundaries, especially so, as the sample is water free.

Figure 1 *c* displays a single-sliced image of a representative sample region, showing all relevant cartilage tissue structures and the subchondral bone, as discussed in §3.1. The centres of the superimposed concentric circles indicate the rotational axis of the sample and are due to persistent ring artefacts (primarily point defects of the scintillator) which were eliminated by filtering the sinogram data. Overall, the tomographic data correspond well with the histological data. The advantage of SR-μCT, e.g. the preserved volumetric representation of the three-dimensional structure, is visualized by three-dimensional rendering in figure 3 (see the electronic supplementary material, movie) and compared with the histological serial sections, which were also rendered. Seventeen out of 20 serially sectioned histological slices were matched with the dimensions of the SR-μCT data (200 single slices). For both renderings, a triangular cut was applied, showing the inside of the sample. All structural details including the visualization of single cells inside the cellular lacunae are observable in the SR-μCT data.

### 3.4. Quantitative SR-μCT

Figure 4 shows the 10 times digitally magnified spatial data of three independent chondrocytes embedded in their lacunae and which have been rendered and recoloured to display three cellular lacunae, with one chondrocyte being occluded from view. For the single separate lacuna and its cell in the foreground, a volume of 1805 μm^3^ has been calculated by summation of thresholded voxels. The SEM/FIB data of a similar lacuna doublet correspond directly to the presented SR-μCT data.

**Figure 5:**
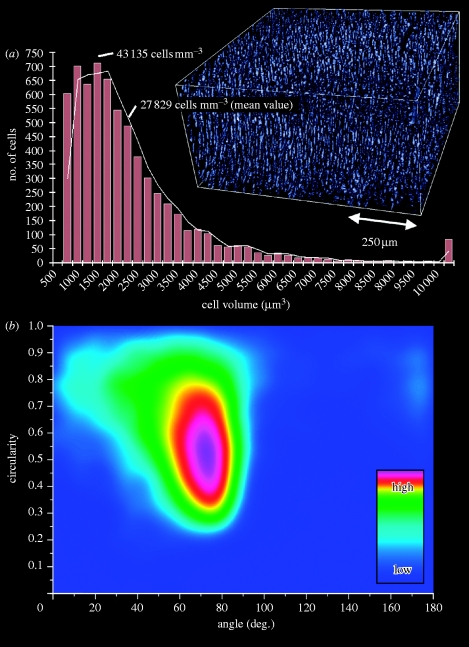
(*a*) Quantification of the cell density in a 0.256 mm^3^ volume of the soft tissue region of articular cartilage. (*b*) Density plot of the lacunae circularity against the orientation (cell number colouring: arbitrary units).

The major benefit of a preserved volumetric sample representation is the possibility to quantify tissue structures. Quantification is demonstrated here in two ways: first, by resolving the cell number and, second, by analysing the shape and orientation of the cells inside the cartilaginous part of the sample, which only consists of the ECM and the embedded chondrocytes. To derive the cell number and consequently the cell density, certain components inside the grey-scale data need to be isolated from the rest. In the presented case, several image optimizations according to table 1 were used prior to data reduction using binarization. These data were analysed for three-dimensional objects (figure 5 *a*), yielding a histogram of the object volumes. Only objects larger than 750 μm^3^ were considered as discussed earlier. For the peak object volume of 1500 μm^3^, this gives a value for the cell density of 43 135 cells mm^−3^ (while for the mean value of 2325 μm^3^, the cell density can be estimated to be 27 829 cells mm^−3^). This corresponds to the literature, which records estimated values for the cell density in cartilage of between 30 000 and 110 000 cells mm^−3^ obtained by confocal laser scanning microscopy (Wong *et al.* 1996) or digital volumetric imaging, as proposed by Jadin *et al.* (2005).

Finally, the object (cell) orientation was determined by plotting circularity against orientation angle (figure 5 *b*). As expected, most cells (lacunae) have an orientation nearly orthogonal to the surface, with a peak at 75° indicating a slightly arched structure. Furthermore, these lacunae are perfectly ellipsoid, having a circularity of 0.5. This actually corresponds well with the typical lacunae in the predominant middle zone of articular cartilage.

## 4. Discussion

In the present study, we have shown that SR-μCT reaches a spatial resolution that enables the tomographic representation of single cells inside tissues without any further metal staining. Although other groups (Mow & Lai 1976; Müller *et al*. 2006*a*,*b*) have already demonstrated that soft tissue structures, such as blood vessels, larger aggregates of mammalian cells or plant cells, can be visualized by means of absorption contrast, often enhanced through heavy metal staining or by phase contrast imaging (Cloetens *et al.* 2006), to our knowledge, the present study demonstrated for the first time the visualization of single mammalian cells without any metal staining. Although individual cells were detectable, quantification was performed on the base of the surrounding lacunae.

In a previous study (Zehbe *et al.* 2007), we have demonstrated that a combined staining with Au/Ag can selectively stain cultivated cells in a porous scaffold and that these cells can be visualized in SR-μCT with the possibility of identifying individual cells. In these analyses, we have also found that the high absorption of the Au/Ag stain can limit the volumetric representation by introducing strong artefacts which might obscure some structural details. Compared with native tissue, the density of this cell-seeded scaffold was very low due to its high porosity, which naturally favoured volumetric separation of cells and the scaffold.

Generally, when applying metal staining on conventional tissue samples, staining occurs primarily through diffusion processes from the outer surface to the inside, which often results in an atomic mass gradient. This gradient, although possibly not limiting in light microscopic or electron microscopic analysis, can obscure some structural details inside the tissue through absorption of the X-ray energy when applying SR-μCT. Therefore, in this work, no metal staining has been used to enhance the low absorption contrast of tissue structures found in native cartilage, which is mainly composed of light elements such as hydrogen, oxygen, carbon and nitrogen. The highest absorption contrast in cartilage tissue is therefore found in the calcified areas of the subchondral bone, which is composed of hydroxyapatite (Ca_10_(PO_4_)_6_(OH)_2_), with a maximum theoretical density of 3.16 g cm^−3^, and calcium being the element of the highest atomic mass (neglecting trace elements).

The SR-μCT data further correspond very well with the data from histology including serial sectioning, showing similar structures in the slice images at approximately the same image resolution. The most limiting factor in serial section imaging is the fact that sectioning losses or slice deformations can make it nearly impossible to completely reconstruct the tissue in three dimensions, thus further preventing in-depth quantification of structural details. Cellular quantification was demonstrated on a 0.256 mm^3^ tissue volume giving a value for the cell density of approximately 40 000 cells mm^−3^. This value corresponds to findings in the literature (Wong *et al.* 1996; Jadin *et al.* 2005). Overall, cellular orientation and shape of the lacunae inside the soft tissue part were as expected, showing a slightly arched structure with mostly ellipsoidal shaped lacunae. The analysis of lacunae orientation and shape can not only help to better model cartilage tissue from a biomechanical viewpoint but also to better understand degenerative diseases of articular cartilage, which are often related to changes in cell and tissue morphology.

One drawback of this study could be attributed to the circumstance that the tissue was not investigated in its native (hydrated) state, but in a dried state after fixation. For the resulting spatial resolution in this experimental set-up, we do not expect a significant difference to the native state apart from the shrinkage, which however may contribute more than 25 per cent according to the work of Kääb *et al.* (1998). Furthermore, shrinkage might be anisotropic, resulting in a complex strain field impacting the tissue morphology. This means that structures are actually larger in the native, hydrated state. Positively, fixation and drying probably made it actually possible to detect single cells in the first place, due to a much enhanced response of a non-watery, dried sample to the phase contrast imaging mode.

In conclusion, conventional histology showed the expected morphology, while SR-μCT gave much superior results considering the possibility to quantitatively analyse the spatial cell density and the structure of the tissue without destroying the specimen. This could be relevant for questions in morphogenesis with respect to physiological processes such as bone and organ development but also with respect to pathological processes such as tumour growth and metastasis.

Thus, finally, SR-μCT offers a very promising tool for the non-destructive three-dimensional structural analyses not only of various tissues under physiological and pathophysiological conditions but also of tissue-engineered cell–scaffold constructs for potential applications in regenerative medicine. In this context, SR-μCT might offer a methodology to validate mechanical stress situations in both native tissues and in tissue-engineered scaffold–cell constructs. Therefore, ongoing experiments of our group are aimed at spatial investigations of different pathophysiological conditions in articular cartilage and at mechanical investigations of cell–scaffold constructs with regard to structural changes under load.

Finally, SR-μCT opens access to a new dimension in the morphological investigation of organs and organ-like tissue constructs, giving important complementary information to the conventional morphological analyses in view of the three-dimensional composition of tissue and engineered tissue such as scaffold–cell constructs.
